# Primary total hip arthroplasty versus internal fixation in displaced fracture of femoral neck in sexa- and septuagenarians

**DOI:** 10.1007/s10195-013-0278-3

**Published:** 2014-01-03

**Authors:** Iftikhar H. Wani, Sidhartha Sharma, Irfan Latoo, A. Q. Salaria, Munir Farooq, Masrat Jan

**Affiliations:** 1 Government Hospital for Bone and Joint Surgery Barzulla Srinagar Kashmir, Srinagar, 190005 Jammu and Kashmir India; 2 Department of Orthopaedics, Government Medical College Jammu, Jammu, 180016 Jammu and Kashmir India; 3 Department of Orthopaedics, ASCOMS, Jammu, 180016 Jammu and Kashmir India; 4 Government Medical College Srinagar, Kashmir, India

**Keywords:** Avascular necrosis, Nonunion, Internal fixation, Arthroplasty

## Abstract

**Background:**

The optimal treatment of femoral neck fracture in the elderly patient is still under debate. In patients aged 60–80 years, the decision between internal fixation and arthroplasty remains controversial. The primary aim of the present study is to evaluate the functional outcome of patients aged 60–80 years with femoral neck fracture treated with total hip arthroplasty or closed reduction and internal fixation. The secondary aim is to evaluate the incidence of nonunion and avascular necrosis in femoral neck fracture in different age groups.

**Materials and Methods:**

We studied 100 patients affected by displaced fracture of the femoral neck from May 2007 through June 2010. There were 60 men and 40 women with mean age of 66 years. Fifty patients were treated with closed reduction and internal fixation with cannulated screws (group A), and the other 50 patients with total hip arthroplasty (group B). Mean surgical time, blood loss, duration of hospital stay, Harris hip score, complications, and need for reoperation were recorded.

**Results:**

Harris hip score was significantly higher in group B at 3-, 6-, 12-, and 18-month follow-up evaluation. The overall complication rate was 28 % in group A and 32 % in group B, which was not statistically significant. A statistically significant difference was found regarding patients who required reoperation in group A (20 %) compared with group B (no one). The average Harris hip score in the internal fixation group was 90.6 and in the total hip arthroplasty group was 93.7, which was statistically significant (*p* < 0.05). Our study showed an increased risk for intracapsular hip fracture developing nonunion with older age.

**Conclusions:**

Primary total hip arthroplasty compared with internal fixation appears to be a reasonably safe method of treating displaced fracture of femoral neck in elderly patients. We also concluded that outcome regarding hip function is generally better after total hip arthroplasty compared with internal fixation.

**Level of evidence:**

Level II-Prospective cohort study.

## Introduction

Femoral neck fractures occur most frequently in elderly female patients. They are uncommon in patients under the age of 60 years, although they can occur at all ages and in both sexes [1]. This is a common skeletal injury accounting for 7 % of admissions to the hospital and occurs with minor trauma in osteoporotic bone of elderly patients and high-velocity trauma in younger patients. The incidence of these fractures has increased due to improved life expectancy with advancements in medical technology and increased vehicular traffic [2].

For more than 100 years, the optimal treatment of femoral neck fracture in the elderly patient has remained under debate [3]. Management of intracapsular hip fractures in the elderly, in general, includes resection of the femoral head and hip replacement [4]. In contrast, for patients younger than 60 years, preservation of the femoral head and anatomic reduction and stable fixation of a femoral neck fracture are the main concerns [5]. However, in patients aged between 60 and 80 years, the decision between internal fixation and arthroplasty remains controversial [4]. In this age group, the optimal treatment should be individualized depending on the fracture pattern and displacement, preoperative ambulation, level of independence, disability, and general health status of the patient [6–8].

The primary aim of the present study is to evaluate the functional outcome of patients aged 60–80 years with femoral neck fracture treated with total hip arthroplasty or closed reduction and internal fixation.

## Materials and Methods

We treated 100 elderly patients with femoral neck fracture in the time period from May 2007 through June 2010. There were 60 men and 40 women with mean age of 66 years (range 60–80 years). The patients were allocated into two groups: group A included 50 patients treated with closed reduction and internal fixation with cannulated hip screws, and group B included 50 patients treated with total hip arthroplasty. Patients of both groups were matched according to age and gender, preoperative medical and physical condition, ambulation ability, and comorbidities (Table 1). During the stated period, many patients with femoral neck fracture were admitted at the authors’ institutions. Inclusion criteria for this study were acute intracapsular femoral neck fracture (Garden types III–IV), age between 60 and 80 years, and independent ambulation before the injury. Of these patients, 100 patients met these criteria and were included in this study. The remaining patients had basicervical/extracapsular hip fracture, bilateral hip fracture, pathological hip fracture, previous ipsilateral hip fracture or hip surgery or decreased hip joint space, significant comorbidities, and intensive care or treatment in medical departments.

**Table 1:** Details of patients included in the study

	Group A (internal fixation)	Group B (total hip arthroplasty)
Mean age (years)	65.16	65.04
Gender (M/F)	26/24	34/16
Side involved (right/left)	22/28	28/22
Garden type IV/III	24/26	36/14
Mechanism of injury		
Simple fall	20	40
Fall from height	16	6
Road traffic accident	14	4

In group A patients, closed reduction and internal fixation with cannulated screws was done; screws were placed within 3 mm of the femoral neck cortex according to Lindequist and Tornkvist [9]. In group B, a cemented femoral prosthesis was inserted. In all group B patients, a posterolateral approach was used [10]. All patients received perioperative antibiotic prophylaxis with second-generation cephalosporin and anticoagulation prophylaxis with low-molecular-weight heparin.

The postoperative mobilization protocol (in patients with internal fixation) included immediate mobilization starting from the second postoperative day with partial weight bearing as tolerated with the use of crutches or a walker for 6 weeks, then full weight bearing. In arthroplasty patients, immediate full weight bearing was allowed as tolerated. Arthroplasty patients were instructed regarding precautions to avoid dislocation of the prosthesis. Length of hospitalization was determined individually in each patient depending primarily on postoperative general medical status and ability to stand or ambulate with a walker. Operative data including duration of surgery, need for blood transfusion, length of hospitalization, and complications were recorded. Postoperative evaluation was done at regular intervals of 3, 6, and 12 months and at the latest examination for the purpose of this study. Functional evaluation was done according to the Harris hip score [11] in both groups. Statistical analysis was done with the paired *t* test for each parameter examined (length of hospitalization, complications, and Harris hip score).

## Results

The mean follow-up was 1.5 years (range 6 months to 3 years). The average pain score in the internal fixation group was 34.64 [mean ± standard deviation (SD) 33.78261 ± 10.4096] and in the total hip arthroplasty group was 41.75 (mean ± SD 41.75 ± 3.193063), which was statistically significant (*p* value <0.05). The mean blood loss for group A was significantly lower [mean 1.4 blood units; 95 % confidence interval (CI) 1.40–1.72] compared with group B (mean 2.6 blood units; 95 % CI 2.60–2.90) (*p* = 0.032).

The mean surgical time for group A was statistically significantly lower (mean 45 min) compared with group B (mean 100 min) (*p* < 0.05). The length of hospitalization was significantly shorter in group A (mean 4.8 days) compared with group B (mean 11.9 days) (*p* < 0.05). None of the patients had complications requiring further intervention at final follow-up in group B (total hip arthroplasty group), whereas four patients had nonunion and six had avascular necrosis in group A, possibly requiring conversion to total hip arthroplasty. The average Harris hip score in the internal fixation group was 90.6 and in the total hip arthroplasty group was 93.7, which was statistically significant (*p* < 0.05) (Table 2).

**Table 2:** Harris hip scores at 3-, 6-, 12-, and 18-month follow-up

Harris hip score	3 months		6 months		12 months		18 months	
	Internal fixation group	Total hip arthroplasty group	Internal fixation group	Total hip arthroplasty group	Internal fixation group	Total hip arthroplasty group	Internal fixation group	Total hip arthroplasty group
Total	75.2	78.1	80.5	83.6	89.6	92.7	90.6	93.7
SD	6.8	4.9	5.6	3.8	4.2	3.6	2.2	2.4
SEM	0.962	0.700	0.792	0.543	0.594	0.514	0.311	0.343
	50	49	50	49	50	49	50	49
*p* value	0.0169		0.0018		0.0002		0.0001	

*SD* standard deviation, *SEM* standard error of the mean, *N* number of patients

We studied the relationship between patient age and incidence of fracture-healing complications after internal fixation of intracapsular fracture. Incidence of both avascular necrosis and nonunion increased with advancing age. Fracture nonunion was less common for Garden type III fracture than for Garden type IV displaced fracture. Incidence of nonunion increased progressively with age, from 2 of 38 patients aged 60–70 years to 2 of 12 patients aged 71–80 years, which was statistically significant. Our study showed an increased risk for intracapsular hip fracture developing nonunion with older age (Table 3).

**Table 3:** Incidence of nonunion and avascular necrosis in femoral neck fracture in different age groups

Age group (years)	Number of patients	Nonunion	Avascular necrosis
60–70	38	2	4
71–80	12	2	2

## Discussion

Surgical treatment of femoral neck fracture is one of the most common procedures performed by orthopedic surgeons. However, the optimal treatment option for displaced femoral neck fracture remains a matter of debate [12–15]. Current treatment options include reduction and internal fixation, hemiarthroplasty, and total hip arthroplasty [5].

There is a growing opinion that outcome would be improved by a more patient-related rather than a strictly diagnosis-related approach; that is, treatment should be based on patient age, functional demand, and individual risk profile [3].

Despite the variety of treatment options, the question of the best treatment for intracapsular hip fracture in the elderly remains open. It has often been claimed that hip function following uneventful healing of a displaced femoral neck fracture will always be better than that following hip arthroplasty [16]. Internal fixation retains the femoral head and the natural hip joint, provided that the fracture unites and the head does not undergo avascular necrosis. The major complications include nonunion and avascular necrosis, which would require a salvage procedure with either hemiarthroplasty or total hip arthroplasty. Following fracture union, second anesthesia may still be required to remove screws that back out.

Numerous studies have provided evidence for better outcomes after arthroplasty when compared with internal fixation in terms of overall functional scores, function of abductor muscles, independent ambulation without walking aids, and quality of life [17–32]. In the present study, we evaluated the treatment of femoral neck fractures in elderly patients using closed reduction and internal fixation with cannulated hip screws compared with hip arthroplasty. The lack of a scientifically strict method of randomization, the treatment decision (albeit not arbitrary) by the treating surgeons, delayed surgical intervention, and shorter follow-up may be considered limitations of this study. However, the number of patients included at the latest examination and the scoring system increase the value of this study.

Although surgical time, operative blood loss, and length of hospitalization were statistically significantly lower in the internal fixation patient group, there were significantly more complications requiring further intervention in the internal fixation group than in the total hip arthroplasty group. Postoperative functional scores up to 18-month evaluation and need for reoperation favored the arthroplasty patient group.

Arthroplasty as a mode of treatment for displaced femoral neck fracture in comparison with internal fixation is associated with a significantly lower risk of revision surgery, at the cost of higher rates of infection, blood loss, and surgical time [16, 18, 20, 26]. Dai et al. [33] recently conducted a meta-analysis comparing the clinical effects of internal fixation with those of arthroplasty for displaced femoral neck fracture in the elderly (≥60 years of age). The combined results of meta-analyses showed no significant difference in mortality at 1 year postoperatively between the two methods. However, compared with internal fixation, arthroplasty could reduce the rate of reoperation and the major method-related complications. Compared with internal fixation, arthroplasty can not only reduce surgical revisions but also decrease the incidence of complications, and does not increase mortality. This meta-analysis shows that there is an evidence base to support arthroplasty as primary treatment for displaced femoral neck fracture in the elderly. Others [34] have also reported that, among people beyond 60 years of age, arthroplasty is associated with better functional outcome, higher health-related quality of life, and greater independence compared with internal fixation. Blomfeldt et al. [16] performed a randomized controlled trial at 4 years comparing internal fixation with total hip replacement for displaced fracture of femoral neck. One hundred and two patients (mean age 80 years) with acute displaced fracture of femoral neck were randomly allocated to be treated with total hip arthroplasty or internal fixation. At 48-month follow-up evaluation, the rate of hip complications was 4 % in patients treated with total hip replacement (THR) and 42 % in those treated with internal fixation, and reoperation rates were 4 and 47 %, respectively. Hip function was significantly better and the decline in health-related quality of life was less pronounced in the arthroplasty group than in the fixation group at 4-, 12-, and 24-month follow-up evaluation. They concluded that, compared with internal fixation, primary total hip replacement provides a better functional outcome for mentally competent elderly patients with a displaced fracture of femoral neck. In the present study, the functional outcome was significantly higher in the arthroplasty group at the 18-month evaluation; however, longer follow-up needs to be carried out to observe persistent statistically significant difference in functional outcome between the two groups.

Although by choosing arthroplasty over internal fixation the surgeon effectively eliminates the risks of avascular necrosis of the femoral head, nonunion, and malunion, a new set of complications is introduced including infection, prosthetic hip joint dislocation, sciatic nerve palsy, femoral stem loosening, thigh pain, and mortality [17–19]. In the present study, in group B, four patients developed superficial wound infection, one patient developed foot drop which recovered partially, one patient developed pulmonary embolism, and there was one death (Table 4). In group A, four patients developed nonunion and six patients avascular necrosis of the femoral head. At the last evaluation, reoperation with total hip arthroplasty was considered necessary in ten patients with nonunion and avascular necrosis (Table 5).

**Table 4:** Complications

Complication	Internal fixation group		Total hip arthroplasty group	
	No. of cases	% of cases	No. of cases	% of cases
Superficial wound infection	2	4	4	8
Urinary retention	2	4	3	6
Bed sore	0	0	4	8
Foot drop	0	0	1	2
Avascular necrosis	6	12	0	0
Urinary tract infection	0	0	2	4
Nonunion	4	8	0	0
Death	0	0	1	2
Pulmonary embolism	0	0	1	2

**Table 5:** Local complications indicating further treatment after initial operation

Group	Complication	No. of patients	Percentage
Internal fixation group	Nonunion	4	8
	Avascular necrosis	6	12
Total hip arthroplasty group	None	None	0

Total hip arthroplasty is currently an accepted treatment option for the active elderly patient with a displaced femoral neck fracture [21]. The longevity of total hip arthroplasty, especially in younger, more active patients, has been questioned. In a previous study [22], 37 patients with mean age of 70 years or younger with no evidence of acetabular disease treated with primary total hip arthroplasty for femoral neck fracture were reviewed. At mean follow-up of 56 months (range 12–112 months), 18 patients (49 %) had undergone or were awaiting revision surgery. In that study, the authors recommended against primary total hip arthroplasty for displaced femoral neck fracture in the younger patient population without preexisting hip disease [22]. Others [23, 24] have concluded that total hip arthroplasty is the best treatment option for active patients with longer life expectancy. Their results are in accordance with the results of the present study regarding the rate of reoperation in patients with displaced femoral neck fracture treated with primary total hip arthroplasty.

In accordance with the literature and our study, we suggest hip arthroplasty for elderly patients with displaced femoral neck fracture and conclude that primary total hip arthroplasty compared with internal fixation appears to be a reasonably safe method of treating displaced fracture of femoral neck in elderly patients. The present study shows that hip arthroplasty compared with internal fixation for treatment of displaced femoral neck fracture significantly reduces the risk of reoperation at the cost of higher superficial infection rate, blood loss, operative time, and length of hospitalization. Furthermore, postoperative function and quality of life as evaluated by the Harris hip score were significantly better in the arthroplasty compared with the internal fixation group up to 18-month evaluation. However, further follow-up records are being collected to determine whether this difference persists at longer follow-up. Our study also shows an increased risk for intracapsular hip fracture developing nonunion with older age.
